# Retraction note: Uterine mast cell tumor: a clinical and cytohistopathological study

**DOI:** 10.1186/s13048-016-0287-y

**Published:** 2016-11-04

**Authors:** Ali Mohammad Bahrami, Fariba Khaki, Shahram Zehtabian, Javad Cheraghi, Mehdi Rashnavadi, Mohammad Reza Hafezi Ahmadi, Mostafa Naderafif, Soheil Javaherypour, Siamak Mohsenzadeh, Ehsan Hosseini, Hamed Masoudi, Mehdi Pourzaer

**Affiliations:** 1 Faculty of Para Veterinary Medicine, Ilam University, Ilam, Iran; 2 Department of Pathology, Faculty of Veterinary Medicine, Tehran University, Tehran, Iran; 3 Department of Microbiology, Faculty of Medicine, Kermanshah University of Medical Sciences, Kermanshah, Iran; 4 Department of Physiology, Faculty of Para Veterinary Medicine, Ilam University, Ilam, Iran; 5 Department of Pathology, Faculty of Medicine, Ilam University of Medical Sciences, Ilam, Iran; 6 Department of Pathobiology, Faculty of Veterinary Medicine, Ferdowsi University of Mashhad, Mashhad, Iran; 7 Student of Veterinary Medicine, Faculty of Veterinary Medicine, Urmia University, Urmia, Iran

The co-Editors-in-Chief and Publisher have retracted this article [1] because the scientific integrity of the content cannot be guaranteed. An investigation by the Publisher found it to be one of a group of articles we have identified as showing evidence suggestive of attempts to subvert the peer review and publication system to inappropriately obtain or allocate authorship. This article showed evidence of plagiarism (most notably from the articles cited [2–6]) and peer review and authorship manipulation.
